# Adaptive deep learning for head and neck cancer detection using hyperspectral imaging

**DOI:** 10.1186/s42492-019-0023-8

**Published:** 2019-11-21

**Authors:** Ling Ma, Guolan Lu, Dongsheng Wang, Xulei Qin, Zhuo Georgia Chen, Baowei Fei

**Affiliations:** 1 Department of Radiology and Imaging Sciences, Emory University, Atlanta, GA 30322 USA; 2 College of Software, Nankai University, Tianjin, 300350 People’s Republic of China; 3 Department of Hematology and Medical Oncology, Emory University, Atlanta, GA 30322 USA; 4 Department of Bioengineering, The University of Texas at Dallas, Richardson, TX 75080 USA; 5 Department of Radiology, The University of Texas Southwestern Medical Center, Dallas, TX 75390 USA

**Keywords:** Hyperspectral imaging, Deep learning, Adaptive learning, Noninvasive cancer detection

## Abstract

It can be challenging to detect tumor margins during surgery for complete resection. The purpose of this work is to develop a novel learning method that learns the difference between the tumor and benign tissue adaptively for cancer detection on hyperspectral images in an animal model. Specifically, an auto-encoder network is trained based on the wavelength bands on hyperspectral images to extract the deep information to create a pixel-wise prediction of cancerous and benign pixel. According to the output hypothesis of each pixel, the misclassified pixels would be reclassified in the right prediction direction based on their adaptive weights. The auto-encoder network is again trained based on these updated pixels. The learner can adaptively improve the ability to identify the cancer and benign tissue by focusing on the misclassified pixels, and thus can improve the detection performance. The adaptive deep learning method highlighting the tumor region proved to be accurate in detecting the tumor boundary on hyperspectral images and achieved a sensitivity of 92.32% and a specificity of 91.31% in our animal experiments. This adaptive learning method on hyperspectral imaging has the potential to provide a noninvasive tool for tumor detection, especially, for the tumor whose margin is indistinct and irregular.

## Introduction

Orophary cancer is a common cancer worldwide and in recent years its incidence increased in a fast pace in both America and Europe [1]. More than half a million patients receive the diagnosis of squamous-cell carcinoma of the head and neck worldwide each year [2]. Survival rate of patients relates directly to the size of the primary tumor at first diagnosis, hence, early detection can be helpful in curing the disease completely. Squamous-cell carcinoma of the head and neck is a complex disease, which can be biopsied for histopathological assessment to make a definitive diagnosis traditionally. That is not only time consuming and invasive, but also subjective and inconsistent [3].

Hyperspectral imaging (HSI) is a technology that can acquire a series of images in many adjacent narrow spectral bands and reconstruct the reflectance spectrum for every pixel of the image [4]. By measuring the reflection and absorption of the lights at different wavelengths, HSI has the ability to simultaneously provide information about different tissue constituents and their spatial distribution from the spectral signature of each pixel in the hyperspectral image [5]. Hence, HSI technique can be applied in the noninvasive detection and diagnosis of cancer, such as breast cancer, gastric cancer, tongue cancer, and so on [6].

Hyperspectral images, known as hypercubes, contain rich information on a wide range of spectra with a high spectral resolution [7], hence, dimensionality reduction, image processing, and machine learning techniques are applied to extract the useful information from the vast amounts of HSI data, and have made many of the advancements in cancer identification: (1) Dimensionality reduction techniques. The principal component analysis [8, 9], tensor decompositions [10], and T-distributed stochastic neighbor approach [11, 12], were to reduce the dimensionality of features in hyperspectral images for compact expression; (2) Image processing techniques. Fourier coefficients [13], normalized difference nuclear index [14], sparse representation [15], box-plot and the watershed method [16], superpixel method [9], markov random fields [17, 18], and morphological method [19], were used for hyperspectral image processing and quantification analysis; (3) Machine learning techniques. Many of the advancements have been done in cancer identification using traditional machine learning classification models, such as linear discriminant analysis [20–26], quadratic discriminant analysis [21], support vector machine [12, 17, 20–22, 27–37], decision trees [22], k-nearest neighbors algorithm [22, 38], k-means [12, 19, 39], naïve bayes [22], random forests [21, 22, 34, 37], maximum likelihood [40], minimum spanning forest [31], gaussian mixture models [41], and semantic texton forest [11], and artificial neural network [33–35, 37], and so on.

However, these technologies require domain-specific knowledge to extract discriminant data to convert suitable features. In contrast to these conventional machine learning techniques, deep learning models can learn representations of data with multiple levels of abstraction, thereby can discover intricate structures in high-dimensional data with very little engineering by hand [42]. Convolutional neural network (CNN) is a type of feed-forward artificial neural network, which has many successes in image recognition, natural language understanding, and medical image analysis [43]. It also can improve the detection and classification performance on HSI [44]. An HSI-based optical biopsy method was proposed using CNN, which could provide multi-category diagnostic information for normal head-and-neck tissue, squamous-cell carcinoma, and thyroid carcinomas [45–47]. A CNN-based modeling framework was introduced for the analysis of hyperspectral images for the detection of head and neck cancer in an animal model [48]. A modified inception-v4 CNN architecture was used to detect the squamous cell carcinoma [49]. In addition, several CNN-based architectures with pixel-wise prediction have shown their efficiency in the segmentation or detection task, such as fully connected networks (FCN) [50], SegNet [51], and U-Net [52]. The U-Net deep neural network was used for the tumor segmentation [53] and the breast tumor detection [54] in hyperspectral images.

Since hyperspectral imagery has the system noise and image artifacts arising from uneven surface illumination, the tumor margin is irregular and unclear. So it is difficult to distinguish a tumor from surrounding normal tissue. In this study, we proposed an automated cancer detection algorithm for highlighting the tumor by adaptive auto-encoder network learning. Auto-encoder is an unsupervised deep neural network that can learn the inherent features and extract the suitable representation from complex data automatically. We involved the auto-encoder network to learn and recognize the depth features of pixels in hyperspectral imagery for the initial cancer detection. Each pixel is assigned a weight according to its classification result. The proposed adaptive auto-encoder learning method is performed on these weighted pixels and is trained to correct the misclassified pixels for the improvement of the detection performance. In this study, we demonstrate the efficiency and effectiveness of the auto-encoder and adaptive deep learning in HSI for head and neck cancer detection in an animal model. The method and experiments are described in the following sections.

## Methods

### HSI system

Hyperspectral images were obtained by a wavelength-scanning CRI Maestro in vivo imaging system. This instrument mainly consists of a flexible fiber-optic lighting system, a solid-state liquid crystal filter, a spectrally optimized lens, and a 16-bit high-resolution charge-coupled device. For image acquisition, the wavelength setting can be defined within the range of 450 to 950 nm with 2-nm increments. Further details can be referred in our previous paper [10, 55].

### The proposed adaptive deep learning method

**Fig. 1:**
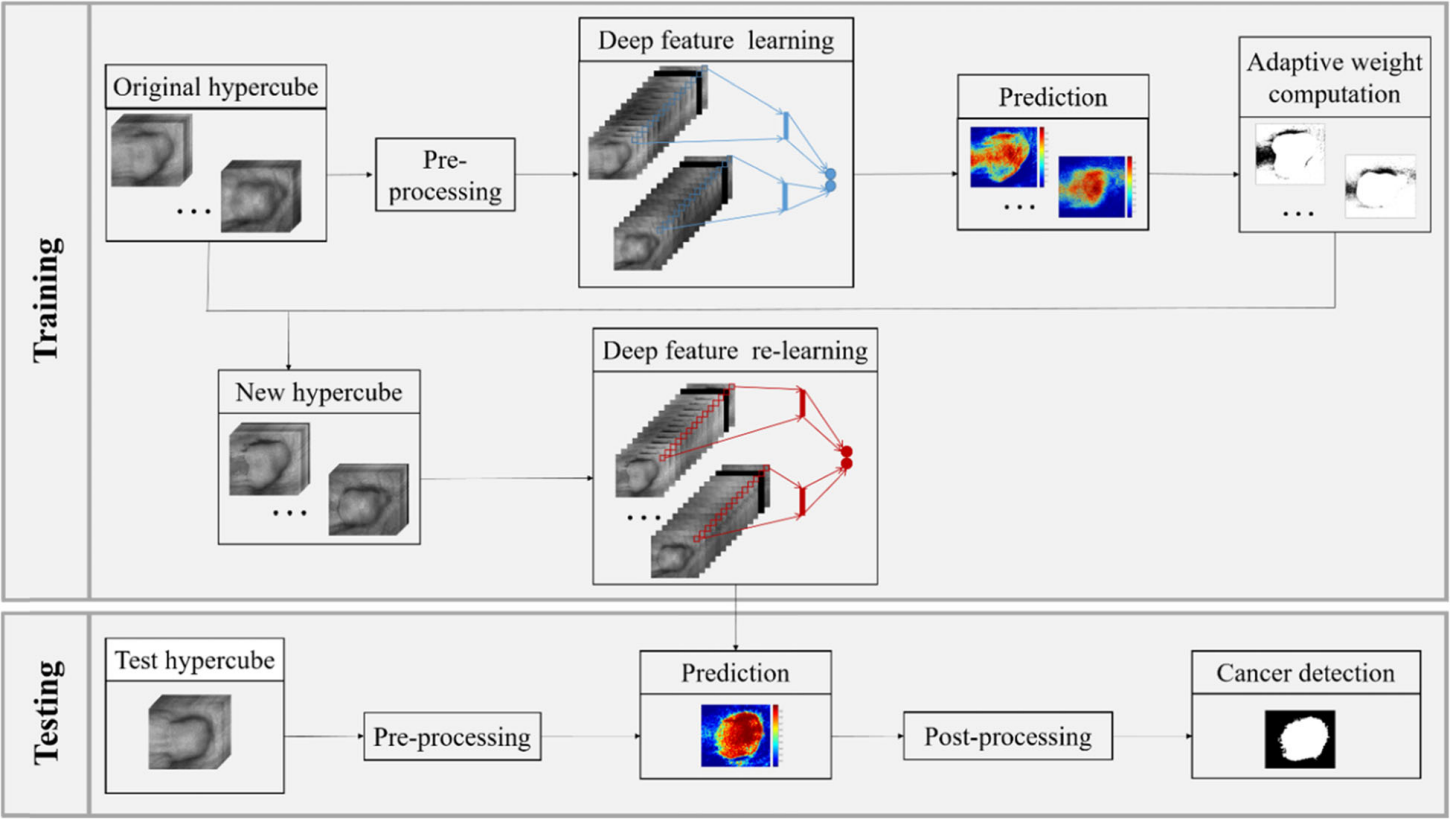
Overview of the proposed adaptive deep learning method for cancer detection with hyperspectral imaging

The proposed adaptive deep learning method for cancer detection on HSI contains four parts: pre-processing, deep feature learning, adaptive weight learning, and post-processing. Figure 1 shows the overview of the method. After the input hypercube is preprocessed, deep feature is extracted and learned for the initial cancer detection. According to the output hypothesis of pixels, the adaptive weights are calculated and the updated hypercube is constructed. The discriminant deep feature is re-extracted and re-learned on the new hypercube. Hence, the re-trained model is adaptive and discriminative. Then, the cancerous tissue in a test hypercube can be identified by the adaptive model, and the detected cancerous tissue is refined by a post-processing step.

#### Pre-processing

The acquired hyperspectral images were saved in a raw format, and correction was made with a white and a dark reference to remove the influence of the dark current and obtain a relative reflectance image. The corrected image, *I*, is calculated by

1 $$ I=\frac{I_{raw}-{I}_{dark}}{I_{white}-{I}_{dark}} $$

where *I*_*raw*_ is the raw image, *I*_*white*_ is the white reference image (100% reflectance) obtained by placing a standard white reference board in the field of view, and *I*_*dark*_ is the dark image (0% reflectance) was acquired by keeping the camera shutter closed. These reference images were used to calibrate hyperspectral raw data before image analysis.

#### Deep feature learning

**Fig. 2:**
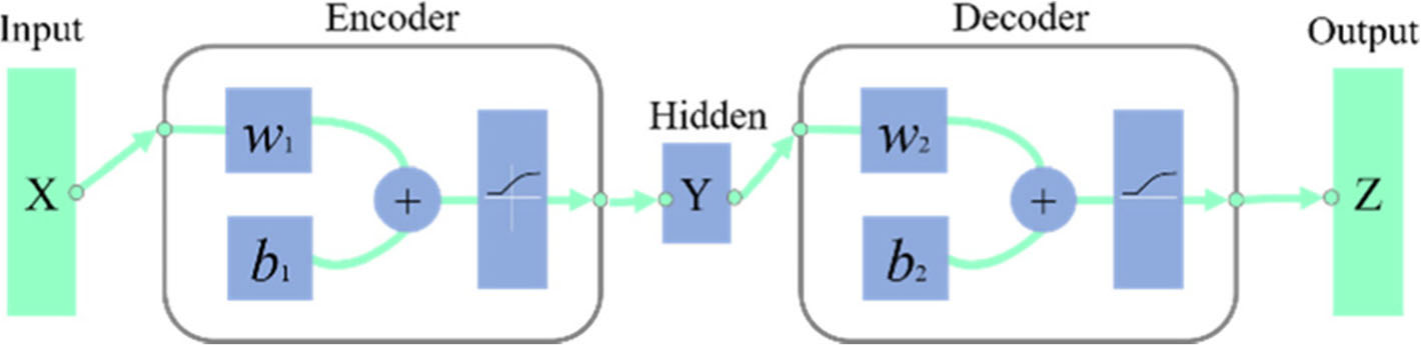
Illustration of an auto-encoder

An auto-encoder is a type of artificial neural network used to learn efficient data coding in an unsupervised manner [56]. It has one visible layer of *k* inputs, one hidden layer of *d* units, one reconstruction layer of *k* units, and an activation function. We illustrate the architectures of an auto-encoder in Fig. 2.

An auto-encoder consists of two parts, the encoder and the decoder. The encoder is to map the input *X* ∈ R^k^ to the hidden layer and produce the latent activity *Y* ∈ R^d^. The decoder is to map *Y* to an output *Z* ∈ R^k^, having the same number of nodes as the input layer, and with the purpose of reconstructing its own inputs *X*. We can get the *Y* and *Z* by

2 $$ {\displaystyle \begin{array}{l}Y=f\left({w}_1X+{b}_1\right)\\ {}Z=f\left({w}_2Y+{b}_2\right)\end{array}} $$

where *w*_1_ and *w*_2_ are the weight of input-to-hidden and the hidden-to-output, and *b*_1_ and *b*_2_ are their bias, *f*(*p*) is the activation function. In our method, it is a sigmoid function like:

3 $$ f(p)=\frac{1}{1+\mathit{\exp}\left(-p\right)} $$

**Fig. 3:**
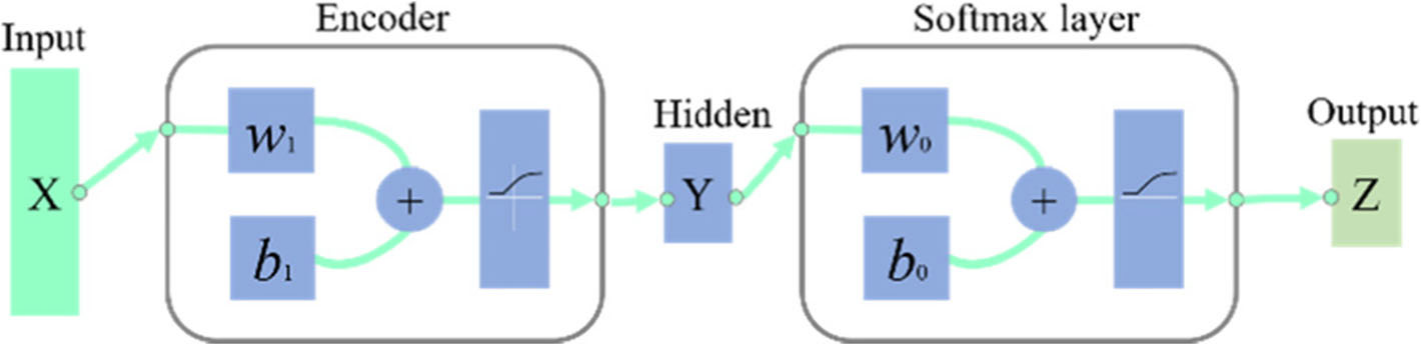
Illustration of an auto-encoder learning for cancer classification

Based on the structure of the auto-encoder, we use the spectral features of each pixel as the input, and train the network by iteratively updating the weights and biases to minimize the loss function of reconstruction error. To improve the robustness of the auto-encoder, in addition to the mean squared error between the input features and the reconstructed features, the L2 regularization and the Kullback-Leibler divergence based sparsity regularization is incorporated into the loss function. The learned feature that lies in the hidden layer is a learned deeper feature. The decoder part is been removed and the softmax layer is added into the network for the classification of cancer and normal tissue. The framework is shown in the Fig. 3.

The auto-encoder network can identify the cancer pixels and healthy pixels. Then we can obtain the initial detection result of cancer and the output hypothesis of pixels.

#### Adaptive weight learning

Because of the characteristics of medical images, the tumor margin is irregular and indistinct. The values of pixels in tumor are similar with those of pixels in healthy tissue. So, it is difficult to distinguish the tumor from its surrounding normal tissue for any learning method. Hence, we highlight the tumor by adjusting its weight adaptively in two aspects according to the misclassified pixels. The misclassified pixels can be divided into two types, the health pixels which are misclassified into cancer (false positives) and the cancer pixels which are misclassified into health (false negatives). The adaptive weight is assigned to each pixel according to its output hypothesis from the initial model, and it can be calculated by

4 $$ weight\left({P}_{ij}\right)=\left\{\begin{array}{c} weight\_ se\left({P}_{ij}\right)=\left\{\begin{array}{c}1,\kern0.5em Pri\left({P}_{ij}\right)= true\left({P}_{ij}\right)\\ {} se(mask),\kern0.5em Pri\left({P}_{ij}\right)=0, true\left({P}_{ij}\right)=1\end{array}\right.\\ {} weight\_ sp\left({P}_{ij}\right)=\left\{\begin{array}{c}\begin{array}{ll}1,& Pri\left({P}_{ij}\right)= true\left({P}_{ij}\right)\end{array}\\ {}\begin{array}{cc} sp(mask),& Pri\left({P}_{ij}\right)=1, true\left({P}_{ij}\right)=0\end{array}\end{array}\right.\end{array}\right. $$

where *mask* is the initial cancer detection, *P*_*ij*_ is the *i*-th and *j*-th pixel in the *mask* and *weight* (*P*_*ij*_) is the weight of *P*_*ij*_. The *weight_se*(*P*_*ij*_) means to focus on the weights of the false negative pixels and the *weight_sp*(*P*_*ij*_) means to focus on the weights of the false positive pixels. The *se* (*mask*) is the sensitivity of the initial cancer detection and the *sp* (*mask*) is the specificity of the initial cancer detection. The *Pri* (*P*_*ij*_) is the prediction of the initial model for the pixel *P*_*ij*_, *true* (*P*_*ij*_) is the true label of the pixel *P*_*ij*_, 0 means the healthy tissue and 1 means the cancer tissue.

Eq. (4) is the definition of weight. We can see that the weight is adaptive because it is related to the local output hypothesis of each pixel and global detection performance. Then the adaptive weights can be used to inform the image, for instance, the misclassified pixels need to change their values in order to become distinguishable from its surrounding tissue. With the help of the weights, the original image can be updated as:

5 $$ hypercube\_ update\left({P}_{ij}\right)= hypercube\left({P}_{ij}\right)\times weight\left({P}_{ij}\right) $$

where *hypercube_update* is the updated hypercube and *hypercube* is the original hypercube, *hypercube_update*(*P*_*ij*_) means the value of *P*_*ij*_ in the updated hypercube and it is the product of the value of *P*_*ij*_ in the original one and the weight of *P*_*ij*_. The main aim of updating the original hypercube is to highlight the tumor most conducive to learning adaptively. Based on the updated hypercube, the deep features can be retrained, and an adaptive auto-encoder network model can be obtained.

Eqs. (4) and (5) show that the initial detected tumor is refined in two ways. If the detected tumor lost some relevant regions, then *weight_se*() could assign the weights to those false negative pixels and the updated image could focus on those pixels. So the new auto-encoder learner on the updated images tends to expand the relevant pixels to improve the sensitivity of tumor detection. If the detected tumor contained some irrelevant regions, then *weight_sp*() could assign the weights to those false positive pixels, and the updated image could highlight those pixels. So the new learner tends to eliminate the irrelevant pixels to improve the specificity of tumor detection. Thus, the pixels classified correctly keep their correct prediction while the pixels misclassified change their values adaptively. Therefore, the adaptive learner heightens the ability to identify the tumor and healthy tissue.

In this paper, the refined type improving the sensitivity or specificity will be determined by experiments according to the detection performance of training images.

#### Post-processing

Since our method is based on the classification of each pixel, the detected tumor may contain some noise and holes. The flood-fill operation is used to fill holes in the segmented binary image and the biggest connected component is chosen as our detected cancer tissue.

## Results

### HSI experiments

All methods were carried out in accordance with the approved Institutional Animal Care and Use Committee protocol (YER-2003103-042918BN) and the relevant guidelines and regulations of Emory University. We acquired the hyperspectral reflectance images from 12 tumor-bearing mice approximately 2 weeks post-tumor cell injection. The reflectance images contained 251 spectral bands and the image size on each spectral band was 390 × 435. Therefore, the data cube collected was a three-dimensional array of the size 390 × 435 × 251. In this study, tumor cells had green fluorescence protein (GFP) signals and thus GFP images were also acquired as the reference standard to evaluate the proposed tumor detection algorithm.

We conducted leave-one-out cross-validation experiments for the tumor detection in hyperspectral images. We take each hyperspectral image as the testing sample in turn, and the 11 remaining samples as the training samples.

### Parameter tuning

**Fig. 4:**
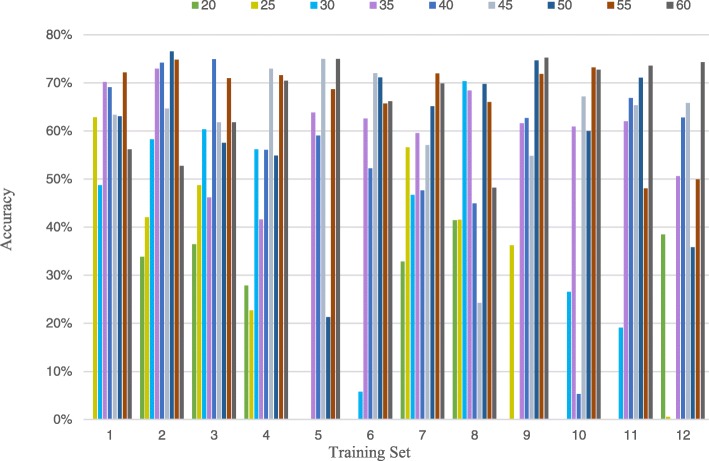
The accuracy in the training process with different dimensions of compressed feature for the 12 mice

The performance of auto-encoder could be affected by its reduced dimensionality. We test the dimension of compressed features from 20 to 60, step 5 on the training set. For each mouse, we train and test on its training set, and calculate the detection accuracy of each training samples, and obtain the average detection accuracy. The results of 12 mice named from #1 to #12 are shown in Fig. 4. The optimal dimensions of compressed feature are 55, 50, 40, 45, 60, 45, 55, 30, 60, 55, 60, and 60 corresponding to the highest accuracy for the 12 mice, respectively.

**Fig. 5:**
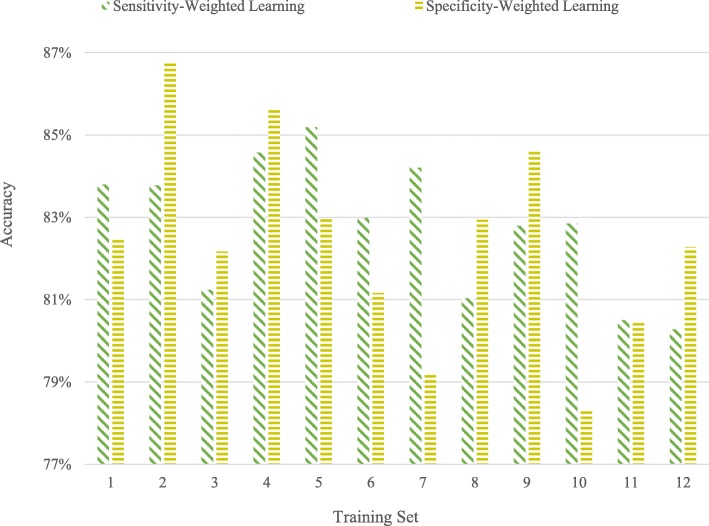
The accuracy in the training process with the adaptive sensitivity-weighted and adaptive specificity-weighted improvement for the 12 mice

Next, we recorded the accuracy obtained by the adaptive sensitivity-weighted learning and specificity-weighted learning on the training set. The initial detected tumor of each mouse in the training set is refined by adjusting its sensitivity-weight and specificity-weight. The average accuracy is shown in Fig. 5. In Fig. 5, we can see that the adaptive sensitivity-weighted learning on the training sets for the mouse #1, #5, #6, #7, #10, and #11 has the higher accuracy than the adaptive specificity-weighted learning, while the adaptive specificity-weighted learning performs better on the other training sets. That shows the refine type we choose depends on the training data. Hence, for the mouse #1, #5, #6, #7, #10, and #11, we will choose the sensitivity-weighted learning for the improvement of cancer detection, and the mouse #2, #3, #4, #8, #9, and #12 will choose the specificity-weighted learning for the improvement.

### Advantage of auto-encoder

**Fig. 6:**
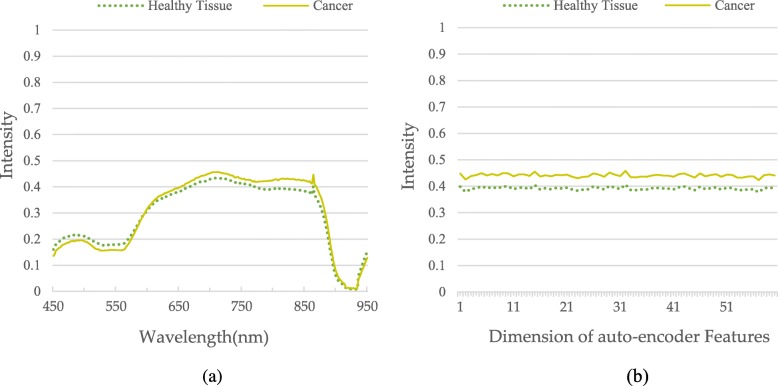
Average spectral values of pixels contained within the cancer and normal regions for (**a**) the original wavelengths and (**b**) compressed features gotten by auto-encoder

To obtain the effective information, the auto-encoder is adopted to learn to compress the 251 wavelengths from 450 to 950 with 2 nm increments into a short feature by extracting the useful characters and ignoring noise. We randomly choose one mouse and use the auto-encoder to obtain the compressed features. Its average spectral values for the cancerous tissue and the healthy tissue of 251 wavelengths and 60 compassed features are shown in Fig. 6. The results in the Fig. 6 show that the difference of the spectral values between the cancerous tissue and the healthy tissue in the compassed features is bigger than that in the original wavelengths. The result demonstrates that auto-encoder can extract the deep features which descripting the discriminate characters for better distinguishing the cancer tissue from the health tissue.

**Fig. 7:**
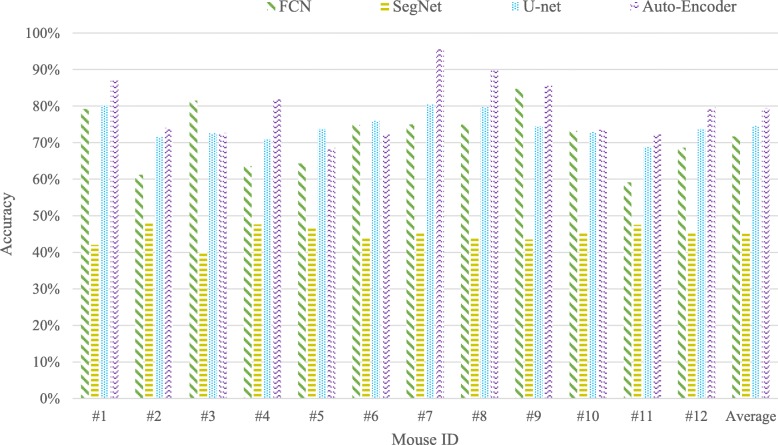
The accuracy of cancer detection for each mouse and their average one for the method FCN, SegNet, U-net, and auto-encoder used in this paper

In addition, we compare the auto-encoder with the CNN-based semantic segmentation neural network, such as FCN [50], SegNet [51], and U-Net [52]. We train the FCN, SegNet, U-Net, and auto-encoder for the initial cancer detection on the HSI, and show the compared results in Fig. 7. In Fig. 7, we can see that the auto-encoder can perform best, so we choose the auto-encoder as our learner, and further improve its detection ability by using the adaptive learning.

### Advantage of adaptive weight learning

**Fig. 8:**
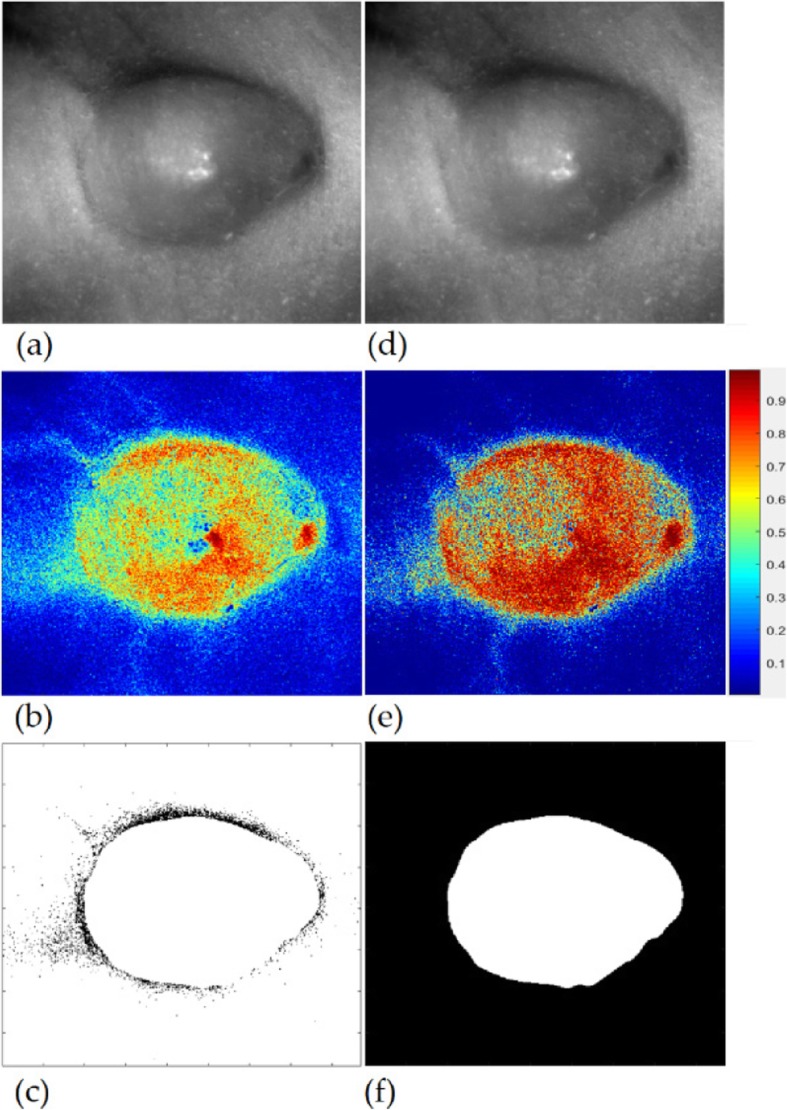
The advantage of adaptive weight learning on the cancer detection. **a**: the original gray image, **b**: the probability image predicted for the original image, **c**: the adaptive weighted image, **d**: the updated image based on the adaptive weight, **e**: the updated probability image predicted for the updated image, **f**: the gold standard of the cancer tissue

To see the advantage of adaptive weight in the detection of cancer tissue, we compare the detection performance of the original learning method and the adaptive weight learning method. The compared results are shown in Fig. 8. Figure 8a is the original gray image by averaging the intensities of 251 spectral bands. It is difficult to identify the part edge of the cancer tissue. The model involved the auto-encoder and neural network learns on the image with 251 spectral bands, and predicts and shows the probability of cancer in Fig. 8b. In Fig. 8b, we can see that most pixels have been classified correctly, that proves the effectiveness of auto-encoder again. However, there are still some misclassified pixels. According to the classification results, the weight of each pixel has been calculated and the weight image is shown in Fig. 8c. In the weight image, we can see that a weight is assigned to each pixel. Based on the weight image, we obtain the new image as shown as Fig. 8d. The new image is tweaked in favor of those misclassified pixels. A new model trained on the new image can achieve better classification performance than that on the original image. The updated prediction result is shown in Fig. 8e. Compared to the gold standard of cancer detection (Fig. 8f), the updated cancer detection performance involved the adaptive weights is better than the original performance, that demonstrates that the adaptive weighted learning is beneficial to improve the accuracy of cancer detection.

### Qualitative results

**Fig. 9:**
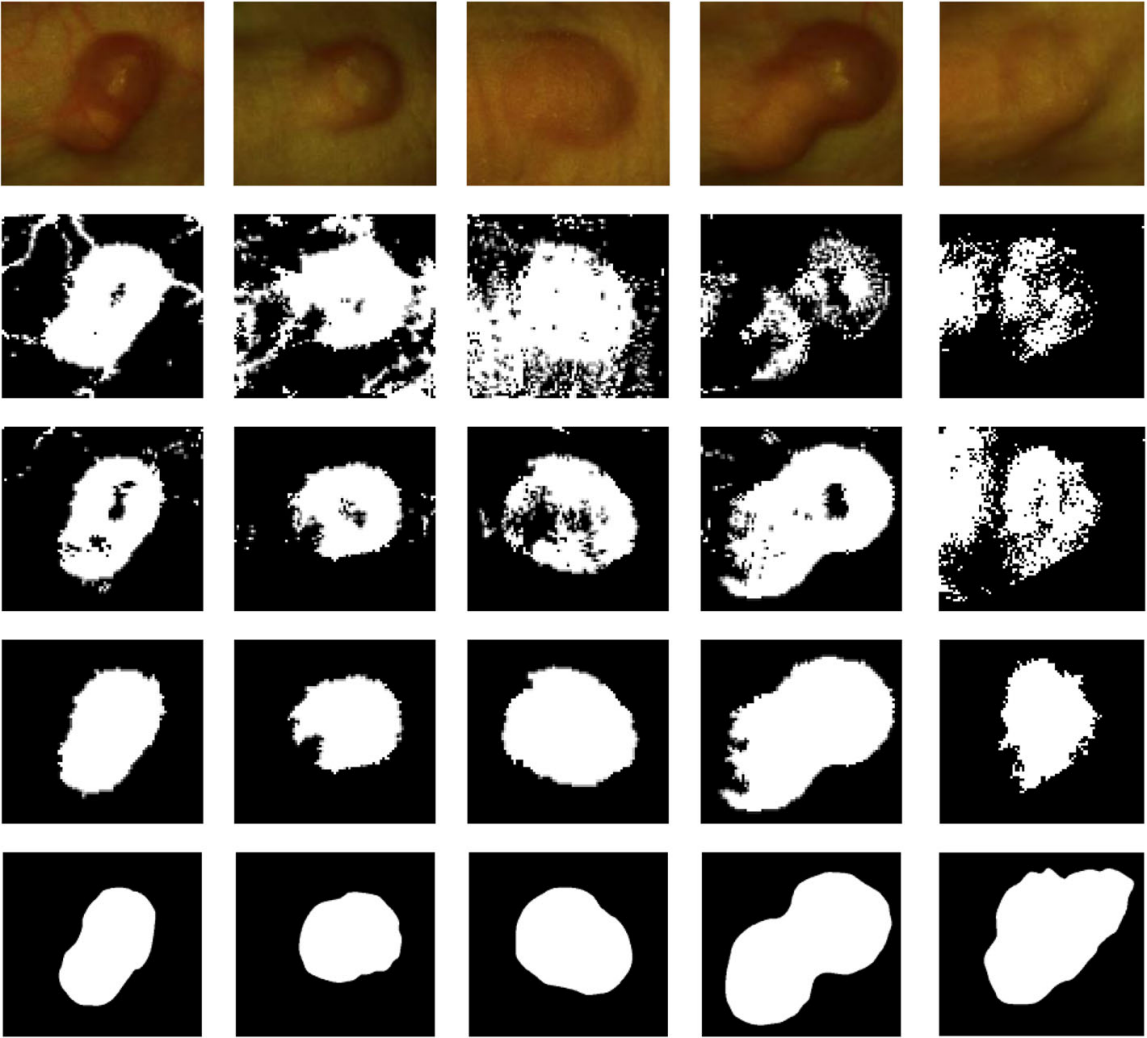
Qualitative evaluation of the cancer detection from five mice. Row 1st: RGB composite images generated from the hypercube, 2nd: The initial detection results, 3rd: The improved results obtained by the adaptive weighted learning, 4th: The final results refined by post-processing, and 5th: The gold standard

We show the qualitative results of cancer detection on five mice in Fig. 9, where the RGB composite images generated from the tumor hypercube are shown in the first row, the initial cancer detection results by the auto-encoder learning are shown in the second row (the white parts mean the cancer tissue while the black parts mean the healthy tissue), the improved detection results by adaptive weighted auto-encoder learning are shown in the third row, the final results refined by post-processing are shown in the fourth row, and the gold standards are shown in the fifth row.

In Fig. 9, we can see that the initial cancer detection results are sensitive to the blood vessel and uneven surface. Since the improved cancer detection method focuses the misclassified pixels with the help of adaptive weight, it is robust to blood vessel, uneven surface and noise, like the detection results of the first and third mouse. Even it can achieve good performance when the tumor has the irregular or unclear margin, like the detection results of the second and fourth mouse. The final detection results refined by post-processing are satisfactory. However, we also can see the performance as shown in the last column is not satisfied. That because the difference between the tumor and normal tissue is too obscure, the intra-similarity between the tumor in the center and the tumor at the edge is much bigger than the inter-similarity between the tumor at the edge and the sounding normal tissue. But we can see that our adaptive auto-encoder method still works better than the auto-encoder method even though when an edge part of the tumor disappears. Hence, our adaptive weight learning is effective.

### Quantitative results

Table 1 provides the quantitative evaluation results of our proposed method for 12 mice. The average sensitivity, specificity, and accuracy of 12 mice are 92.32%, 91.31%, and 91.33%, respectively. These results confirm that our method is effective. Based on the above results, we know that the bad performances of the last two mice shown in Table 1 were caused by the tissue artifacts and the invasion of surrounding tissue. Although our method received unsatisfied results when part of tumors disappear, it could obtain above 80% accuracy. If we remove the two mice, the average sensitivity, specificity, and accuracy, will be 94.72%, 92.26%, and 92.97%, respectively.

**Table 1:** The performance of cancer detection for 12 mice

Mouse ID	Sensitivity (%)	Specificity (%)	Accuracy (%)
1	99.50	94.49	95.52
2	90.67	92.48	91.78
3	93.85	96.48	95.98
4	99.77	90.89	93.13
5	93.16	98.08	96.79
6	98.91	95.54	96.27
7	96.13	94.20	94.50
8	95.49	90.03	92.28
9	91.67	86.16	87.80
10	88.10	84.22	85.63
11	60.84	99.63	85.79
12	99.78	73.53	80.52
Average	92.32	91.31	91.33

## Discussion

In this study, we proposed automated detection method for head and neck cancer using the adaptive deep learning on hyperspectral imagery in an animal model. Auto-encoder network model is involved to extract the deep features from a hyperspectral imagery with size of 390 × 435 × 251 and distinguish the cancerous tissue from its surrounding normal tissue. Because of the noise and uneven surface and so on, the detected cancer region is not satisfactory. To improve the initial performance and obtain a complete tumor, the adaptive auto-encoder network model is proposed, which focus on the misclassified pixels and enhance to learn for the misclassified pixels. The method is shown to classify the tumor region with high sensitivity, specificity, and accuracy.

Since reflectance hyperspectral images contain 251 spectral bands from 450 to 950 nm with 2 nm increments, and each hyperspectral image contains over millions of reflectance spectral signatures, it is difficult to extract discriminant features from the huge data by hand. Deep learning methods can learn features by building high-level features from low-level ones and automatically discover the features needed for cancer detection. The auto-encoder is an unsupervised deep neural network that tries to denoise the inputs automatically by finding the latent representation from which to reconstruct the original input, hence it is especially suitable for describing the hyperspectral data. As shown in the Fig. 6, the extracted features by auto-encoder method can better distinguish the cancerous tissue from the non-cancerous tissues. In addition, the auto-encoder can achieve higher accuracy compared with the other neural network models, hence, the auto-encoder is used as the learner for the initial cancer detection.

Although auto-encoder can extract the useful information for detecting the cancer tissue, the blood vessel or uneven surface make it difficult to identify the complete tumor from the normal tissue. Since blood vessel or uneven surface could misrepresent the tissue structures, neither the traditional classification methods nor the deep learning methods could detect the tumor with high accuracy by learning the intensity or the distribution of intensity. The proposed adaptive auto-encoder network can predict the misrepresented tissue structures into their true classes by adaptively weighting those misclassified pixels, and thus greatly improve the performance of tumor detection, as shown in Figs. 7, 8, 9 and Table 1. Under the same conditions, our proposed method performed better than the traditional classification methods [10, 31], and the CNN based deep learning method [45, 50–52]. However, we obtain unsatisfied performance on some mice. In the next work, we plan to improve the cancer detection performance on those special images. We will learn the deep feature by iteratively updating the sensitivity-weights and specificity-weights until convergence to overcome the effects of noise and artifacts.

The automatic detection algorithm was written and run in MATLAB on Intel Core 2.60GHz CPU with 16GB of RAM. The time for normalization, deep feature extraction, cancer detection, post-processing is about 0.1 s, 2.8 s, 3.2 s, and 0.02 s, respectively. The total running time is about 6 s for per hyperspectral image. It greatly improved the efficiency of cancer detection compared with the method [31] using 45 min. This automatic cancer detection method can be implemented in real time if involving the multi-thread, GPU acceleration or parallel programming.

## Conclusions

In this study, an adaptive deep learning framework was proposed and validated for head and neck cancer detection using HSI in an animal model. This algorithm extracted the deep feature of hyperspectral images for reducing the dimensionality effectively and better characterizing the cancerous tissue. The adaptive weight learning could improve the cancer detection performance by focusing on harder-to-classify pixels. In the head and neck cancer mouse model, the proposed cancer detection method was able to obtain a high sensitivity and specificity. The results demonstrated that the HSI combined with deep learning technique may enable accurate and fast detection of cancers in a noninvasive manner and may provide a promising tool for future clinical applications.

## Data Availability

The datasets used and/or analyzed during the current study are available from the corresponding author on reasonable request.

## Abbreviations

CNN: Convolutional neural network
FCN: Fully connected networks
GFP: Green fluorescence protein
HSI: Hyperspectralimaging
